# A comparative study of sonographic and clinical parameters in patient with upper trapezius muscle trigger point following dry needling and intramuscular electrical stimulation: a randomized control trial

**DOI:** 10.1186/s12998-024-00567-8

**Published:** 2025-04-14

**Authors:** Monavar Hadizadeh, Abbas Rahimi, Meysam Velayati, Mohammad Javaherian, Farokh Naderi, Abbasali Keshtkar, Jan Dommerholt

**Affiliations:** 1 Department of Physiotherapy, School of Rehabilitation, Shahid Beheshti University of Medical Sciences, # Damavand Ave, Tehran, 16169-13111 Iran; 2 Department of Radiology, Akhtar Orthopedic Hospital, Shahid Beheshti University of Medical Sciences, Tehran, Iran; 3 Department of Physiotherapy, School of Rehabilitation, Tehran University of Medical Sciences, Tehran, Iran; 4 Department of Radiology, Iran University of Medical Sciences, Tehran, Iran; 5 Department of Health Sciences Education Development, School of Public Health, Tehran University of Medical Sciences, Tehran, Iran; 6 Bethesda Physiocare, Bethesda, MD USA; 7 Myopain Seminars, Bethesda, MD USA; 8 Department of Physical Therapy and Rehabilitation Science, School of Medicine, University of Maryland, Baltimore, MD USA

**Keywords:** Trigger point, Myofascial pain, Intramuscular electrical stimulation, Dry needling, Ultrasound imaging, Upper trapezius

## Abstract

**Background:**

The most common cause of muscle pain is myofascial pain syndrome. Myofascial pain syndrome caused by sensitive areas called trigger points (TrP). Some physiotherapy modalities have acceptable effects for this disorder, but it is necessary to check the effects of placebo, appropriate dose, and long-term effects for each intervention. The aim of this study is to investigate the effect of intramuscular electrical stimulation (IMES) compared to dry needling (DN) on sonographic and clinical parameters in upper trapezius muscle TrP.

**Methods:**

This is a randomized, single-blind control trial. The study period was from December 2, 2020, to April 10, 2021. Thirty volunteer patients with active upper trapezius TrP were randomly allocated into two groups: (1) IMES, (2) DN. Participants received interventions in three sessions. Primary outcome measurements were neck range of motion (ROM) and TrP circumference. Secondary outcome measurements were pain by visual analog scale (VAS), pain pressure threshold (PPT), disability, TrP longitudinal and transverse diameter, TrP stiffness, and muscle blood flow by vascular resistance index (RI). All outcome measurements were evaluated before, after, and one month after the intervention. If the data were normal, the repeated measure ANOVA test was used; if data were not normal, the Friedman test and the Kruskal-Wallis test was used. A significance level of 0.05 has considered.

**Results:**

ROM increment was significantly more in the IMES group. TrP circumference decrement was significantly more in the IMES group. VAS changes did not show significant difference between two groups. The PPT improvement was significantly more in the IMES group. Disability changes were not significant. Longitudinal diameter changes were significantly more in the IMES group. TrP stiffness changes were not significant. The vascular RI decreased significantly in IMES group.

**Conclusion:**

It seems that both IMES and DN have promising effects for improving upper trapezius TrPs. However, IMES is more effective in some clinical and ultrasound parameters. In order to investigate the effects of this intervention more precisely more studies are necessary.

**Trial registration:**

This study was prospectively registered at Iranian registry of clinical trials (IRCT: IRCT20170616034567N2).

**Supplementary Information:**

The online version contains supplementary material available at 10.1186/s12998-024-00567-8.

## Background

Musculoskeletal pain is one of the most common reasons of chronic widespread pain and one of the main concerns of societies [1]. One of the common causes of muscle pain is myofascial pain syndrome (MPS), which affects the majority of the population and reduces people’s overall sense of well-being [2]. Myofascial pain syndrome features sensory, motor, and autonomic symptoms caused by sensitive areas called trigger points (TrP) [3–5]. The most common definition of a TrP is “a sensitive point in a taut band of skeletal muscle that becomes painful with pressure, stretch or contraction and causes referred pain” [4, 5]. Trigger points are very common and occur at least one time during life for most people. The syndrome may involve any muscle [3].

The upper trapezius (UT) muscle is frequently affected by MPS and several pain conditions are associated with UT muscle TrPs, including migraine, neck pain, chronic tension headache, breast cancer, and shoulder disorders [6–10]. The treatment may include interrupting the pain cycle by inactivating TrPs, manipulation, compression, physiotherapy modalities, muscle stretching, and injections. Some physiotherapy modalities and manual therapy have acceptable clinical evidence, but in research, it is necessary to check the effects of placebo, appropriate dose, and long-term effects [11–17]. A typical intervention used in clinical practice is dry needling (DN), which is considered an effective treatment option for TrPs [18], although more research is needed to check this intervention’s effectiveness and its long-term effects. The American Physical Therapy Association (APTA) defined DN as “skilled intervention using a thin filiform needle to stimulate TrPs, musculature and connective tissue for the management of neuromusculoskeletal disorders” [19]. Eliciting a local twitch response (LTR) during DN will increase the effectiveness of this intervention [20–22]. Intramuscular electrical stimulation (IMES) is a recently introduced therapeutic method. The efficacy of IMES has been evaluated in a limited number of studies with relatively promising results [23–28]. Considering that this treatment can lead to more muscle micro contraction, it may be an effective intervention for TrPs [29]. To date, only clinical and subjective outcome measurements have been used to examine specific changes in individuals with TrP. None of the previous studies have investigated the effects of IMES compared to DN using an objective method. Unfortunately, no universally accepted objective diagnostic criteria exist, and TrP diagnosis is made manually [30]. Although several studies showed poor inter-rater reliability of manual palpation, others supported much better validity and reliability, which may be related to the examiner’s experience, the depth of the muscle, as well as to the active or latent TrP [31–44]. Nevertheless, the accuracy of the TrP diagnosis may be improved with more objective methods, such as sonography [45–47]. In addition, previous studies have reported that ultrasonography is a reliable procedure for assessing the UT muscle characteristics in patients with MPS as an objective method for assessment [47].

This study aimed to compare the effects of intramuscular electrical stimulation versus dry needling on trigger points of the upper trapezius muscle on clinical symptoms and sonographic parameters, including morphological and hemodynamical properties of trigger point and upper trapezius muscle. Sonographic parameters have been used as an objective outcome to detect the presence and measurement of trigger point changes after interventions along with clinical outcomes.

## Methods

### Study design

A randomized, single-blind clinical trial was conducted to compare the therapeutic effects of IMES and DN. The study design was approved by the Shahid Beheshti University of Medical science Ethics Committee (IR.SBMU.RETECH.REC.1399.480, dated: August 9, 2020), and the study was prospectively registered (IRCT: IRCT20170616034567N2). The study period was from December 2, 2020, to April 10, 2021. The study setting was Faculty of Rehabilitation, Shahid Beheshti University of Medical Science, Tehran, Iran. The study was conducted according to the Consolidated Standards of Reporting Trials (CONSORT) statement and included the CONSORT checklist.

### Participants

Thirty patients have volunteered for the study and were allocated by a balance block randomization method [48] (which was performed by someone who was not involved in the study process) to one of two groups: the IMES group (11- women, 4-men) and the DN-group (13- women, 2-men). Block randomization is a commonly used technique in clinical trial design to reduce bias and achieve balance in the allocation of participants to treatment arms [48]. The sample size was calculated based on the work of Hadizadeh et al. (2017), who compared IMES vs. placebo based on cervical lateral flexion range of motion (ROM) [28]. Considering the probability of loss during follow-up (20%), the number of participants required to attain a power of 0.9 and a bilateral α _level of 0.05 for analysis of variance (ANOVA) with repeated measures was calculated to be 15 per group.

Before testing, all subjects signed a written informed consent. The participants were invited to the study by using an advertisement in Shahid Beheshti University of Medical Science, Tehran, Iran, and using virtual space. The inclusion criteria were: (1) patients aged between 18 and 50 years, (2) having active TrP in the UT muscle according to the following criteria [49]: (a) the presence of a taut band; (b) a hypersensitive spot within that band; (c) referred pain, 3) confirmed presence of a TrP in the form of a hypoechoic zone in a B-mode image using the sonographic technique by an expert musculoskeletal radiologist [50], 4) having pain associated with the UT muscle TrP greater than 3/10 on a Visual Analogue Scale (VAS), which is considered moderate pain [51]. A physiotherapist with more than 5 years of experience in identifying TrPs determined the presence of TrP. Exclusion criteria were: (1) muscle diseases such as myopathy, (2) fibromyalgia, (3) malignancy or being disposed to infection, (4) neurological disorders, (5) any history of neck and shoulder surgery or trauma, (6) any medical treatment or physical therapies for UT muscle TrP during the last month, (7) history of a poor response to acupuncture or DN [52], (8) significant fear of DN [52], (9) vascular disease [52], (10) diabetes [52], 11) history of migraine [52].

### Randomization

Balance block randomization was performed by someone who was not involved in the study process. Wrapped opaque envelopes were prepared with the assigned treatment. The envelopes were numbered consecutively.

### Intervention

After clinical assessment, an expert radiologist with more than 10 years of experience in musculoskeletal sonography recorded sonographic images. The same experienced physiotherapist who identified the TrP, performed both interventions. Interventions were performed in three sessions during one week. After the last session, the therapist and radiologist performed the post-intervention assessment.

### Intramuscular electrical stimulation

**Fig. 1:**
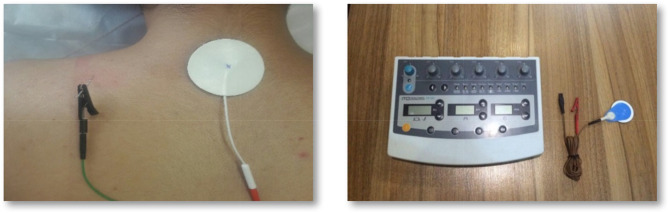
IMES intervention procedure

Patients were lying in a prone position with the arms adjacent to the body. A solid filament needle (0.30 mm diameter and 50 mm length) was inserted into a TrP [53]. Next, the cathode electrode was attached to the needle, and an anode pole was placed on the spinous process of the C7 by an adhesive electrode. A burst current with a frequency of 2 Hz and a pulse width of 200 microseconds was applied for 10 min to the active TrP (Fig. 1). The intensity was increased until a painless contraction was visible. The device used in this study (ES-160 by ITO (Japan)) was calibrated before the study (Fig. 1).

### Dry needling

**Fig. 2:**
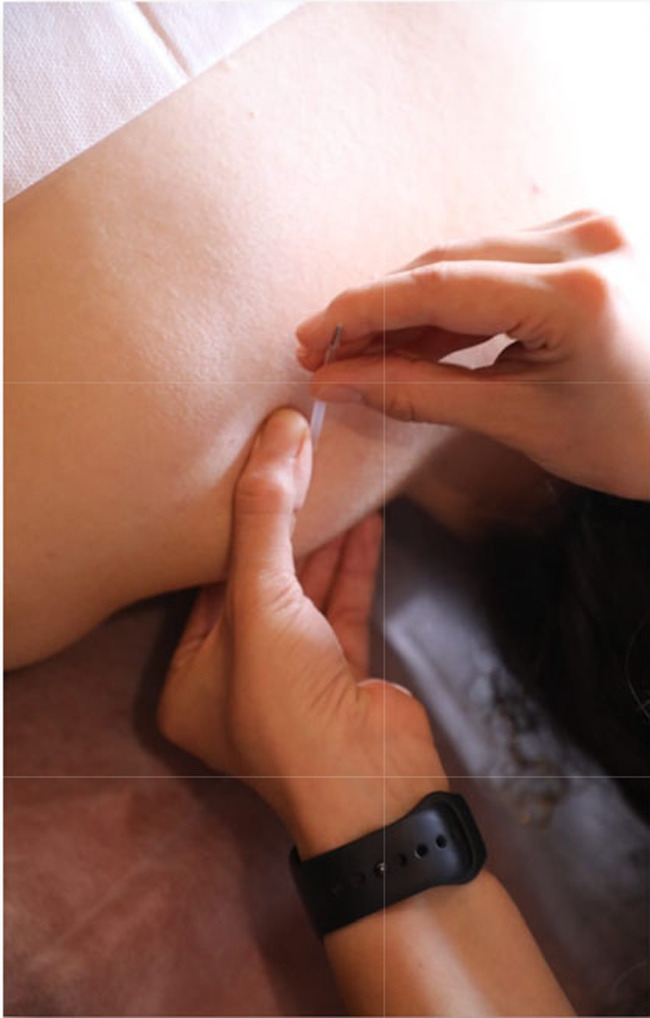
DN intervention procedure

Patients were lying in a prone position, and DN was performed on TrP with the same procedures as the IMES group to provoke LTR. The needle was moved forward and backward until LTRs were no longer seen after ten repetitions of needle movement (Fig. 2).

### Outcome measures

All outcome measures were assessed at baseline, after treatment sessions, and one month after. The radiologist was blinded to the group allocation.

### Primary outcome measures

The primary outcome measures were cervical lateral flexion ROM (clinical) and TrP circumference (CIR) (sonographic).

#### Cervical lateral flexion ROM

A goniometer was used for ROM measurements. The goniometer axis was placed on the C7 spinous process. The fixed arm of the goniometer was perpendicular to the ground, and its flexible arm was located in the posterior midline of the skull. Each subject performed the movement to the opposite side, and the degree of movement was recorded [54]. The excellent reliability of this tool for measuring active ROM has already been reported [55].

#### TrP CIR

**Fig. 3:**
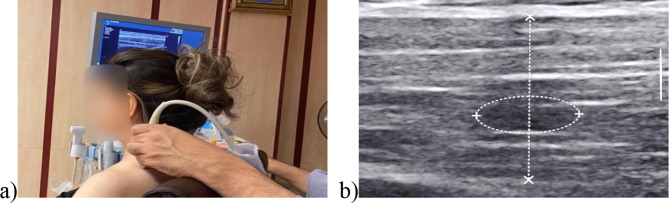
(**a**) participants position during sonography; (**b**) TrP CIR in UT muscle gray scale image

Ultrasound images of the UT muscle were recorded using a linear probe ultrasound device with a 5 to 18 MHz frequency (Aixplorer Supersonic Imagine, France, 2017). To record the ultrasound images, participants were seated with the head in the neutral position, with 90-degree flexion of the knee and hip while the hands rested on the armrest. The linear probe was placed perpendicular to the UT muscle, parallel to the muscle fibers. The probe was moved to the point where the muscle fibers were parallel in the gray-scale image (Fig. 3-a). Gray-scale images were recorded, and TrP CIR was measured on a frozen image (Fig. 3-b). Ultrasonography is a reliable technique for measuring the UT muscle characteristics in patients with MPS [47, 50].

### Secondary outcome measures

The secondary outcome measures were pain intensity, pain pressure threshold (PPT), neck disability index (NDI) (clinical) and TrP longitudinal and transverse diameter, TrP stiffness, and blood circulation (sonographic).

### Clinical parameters

#### Pain intensity

A VAS was used, which is a well-established and validated self-report to measure the intensity of pain. Subjects marked their pain level on a 10 cm (100 mm) line considering that 0 indicated no pain and 10 meant the most imaginable pain. The VAS showed moderate to good reliability (intraclass correlation coefficient 0.60 to 0.77) for assessing disability in patients with chronic musculoskeletal pain [56].

#### Pain pressure threshold

PPT was evaluated using a digital algometer (FG-8020 -Taiwan). PPT is defined as the minimum pressure that induces pain or discomfort. The pressure was applied slowly to the level that participants could detect pain. Two consecutive tests were performed on the active TrP of the UT muscle at 5 min intervals. Intra-evaluator reliability is high in the UT muscle [57].

#### Neck disability index

The validated and reliable Persian version of the NDI was used to assess the disability. The NDI is a valid tool for measuring pain and self-assessment of cervical disability. The NDI is formed of 10 questions correlated to daily functional activities. Each section (intensity of neck pain, personal care, weight lifting, reading, headaches, ability to concentrate, work capacity, driving, sleep, and leisure activities) offers six possible answers representing six levels of progressive functional capacity. It is scored from 0 to 5 (0 = no disability, 5 = total disability). The total score is a percentage of the maximum possible [58–60].

#### Sonographic parameters

To be sure that a same trigger point is checked during the assessment sessions. The point was marked and the distance of the point from the spinous process of the 7th cervical vertebra and from the scapular process was measured and these measurements were recorded for each person.

#### TrP longitudinal and transverse diameter

To measure the diameter of a TrP, a gray-scale image was recorded with the same procedure as mentioned before, and the TrP longitudinal and transverse diameters were measured on frozen images (Fig. 3-b).

#### TrP stiffness

**Fig. 4:**
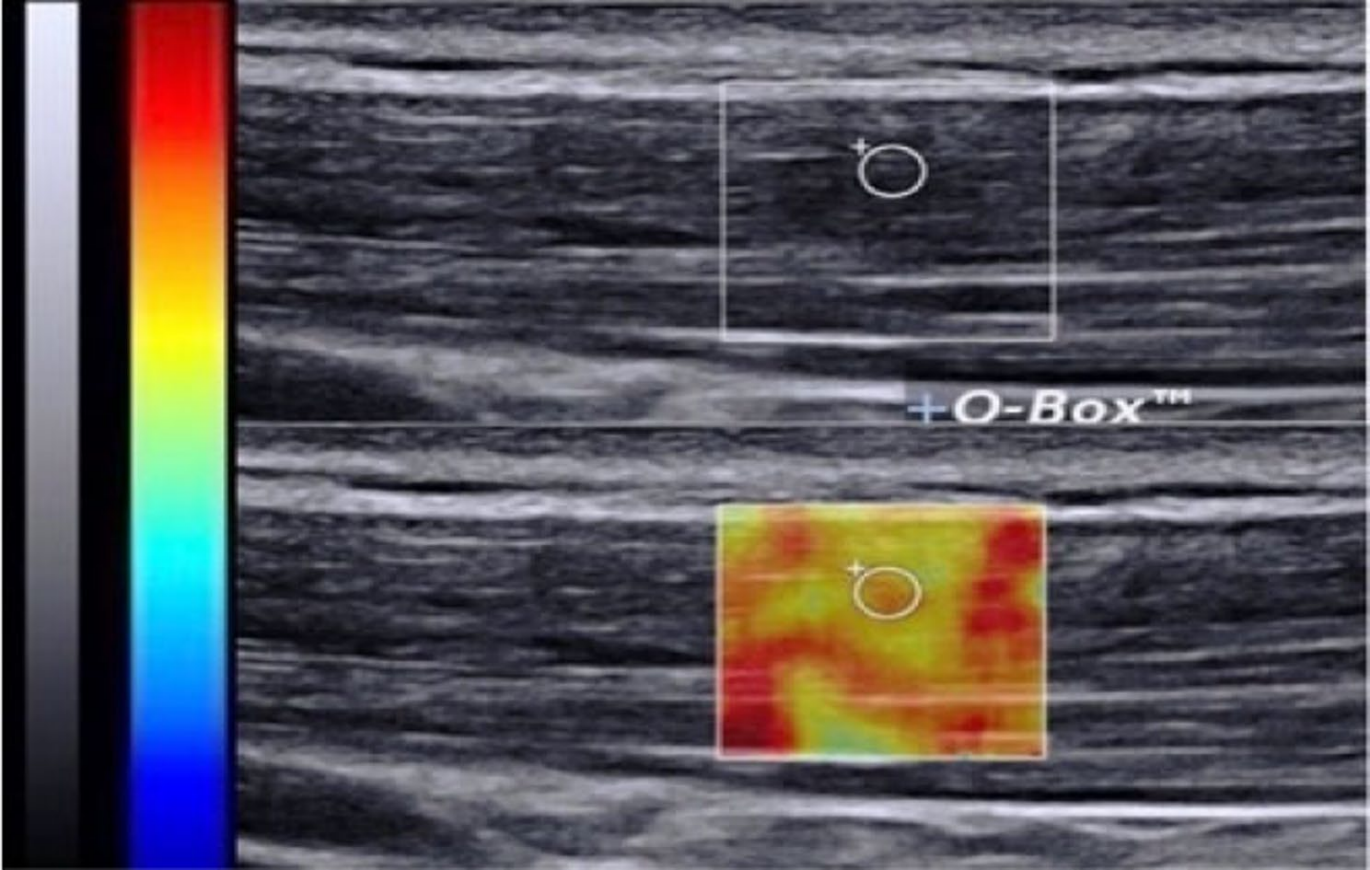
TrP stiffness in UT muscle gray scale image

To measure TrP stiffness, gray-scale imaging was performed at the same method as the previously mentioned. The color-coded image was superimposed on a gray-scale image during imaging. In this design, softer parts were coded as blue, while stiffer parts were coded as red. With the proper image selection, a circle region of interest (ROI) with a size of 4 mm^2^ was adjusted on the center of the TrP on the frozen image. Muscle stiffness was calculated automatically by the sonography device software as shear-wave elastography. This is an ultrasound elastography technique that uses shear waves to measure tissue stiffness quantitatively. In this procedure shear waves travel perpendicularly through muscle to generate a shear wave that is measured by assessing muscle tissue displacement [61] (Fig. 4).

#### Blood circulation

**Fig. 5:**
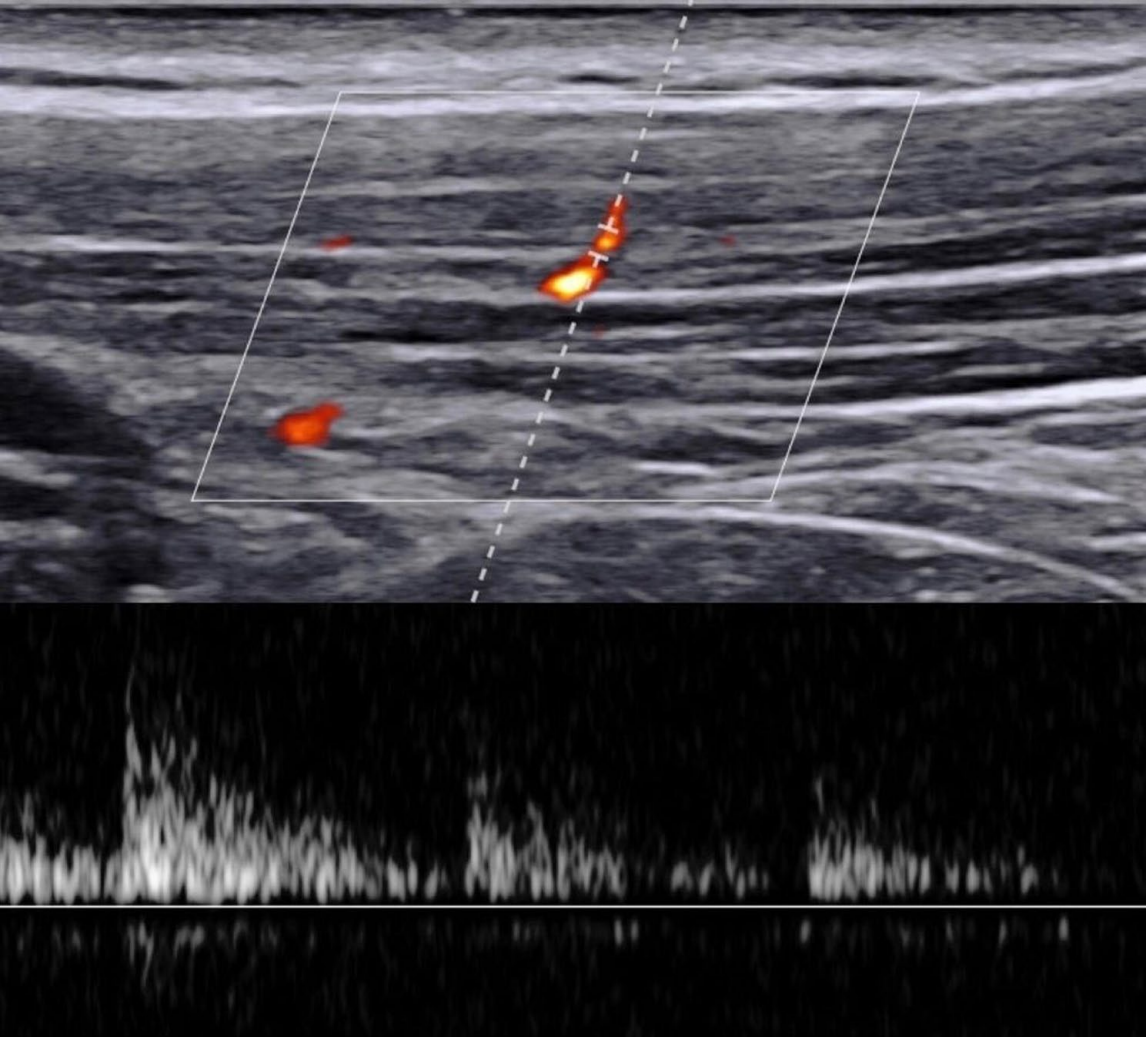
RI measurement in the TrP vicinity

Color Doppler ultrasonography was used to scan UT muscle circulation, which is a reliable technique [47]. The Doppler waveforms were used to determine each cardiac cycle peak systolic velocity (PSV), end diastolic velocity (EDV), and resistance index (RI). The RI is a ratio obtained by the difference between PSV and EDV to the PSV. Lower RI is a sign of higher blood circulation. For this study setup, these parameters were calculated in the arterioles or arteries found in the TrP vicinity of the UT muscle in the images (Fig. 5).

### Statistical analysis

The statistical analysis was performed using STATA Statistics version 13.0 (which was performed by someone who was not involved in the study process). A Kolmogorov–Smirnov test was used to test normality. All the variables presented a normal distribution, excluding pain intensity, PPT, and stiffness. A descriptive analysis included the mean ± SD was selected to summarize the outcomes in the 3 measurements in both groups (IMES group and DN group). The demographic data were analyzed by descriptive statistics, and measures are presented as the mean ± SD.

A repeated-measures ANOVA was performed for all normal variables to analyze the between-groups (IMES group and DN group) considering the size of the same variable (before the intervention) as covariance, and the within-group (Pre, post, and one month after treatment). When differences were established, a post-hoc Scheffe multiple-comparisons test was applied. The point estimates of effects were reported as MD with a 95% confidence interval (CI), standardized mean difference (SMD) with 95% CI analyzed using Glass’s Delta. The statistical significance level was determined as *p* < 0.05. We considered 0.2 − 0.49, 0.5 − 0.79, 0.8–1.19, and > 1.2 values as small, moderate, large, and very large effect size, respectively. Non-parametric tests were used to analyze non-normal variables. Friedman test was used to analyze within-group effects, and Kruskal–Wallis test was used to analyze between-group comparison.

## Results

**Fig. 6:**
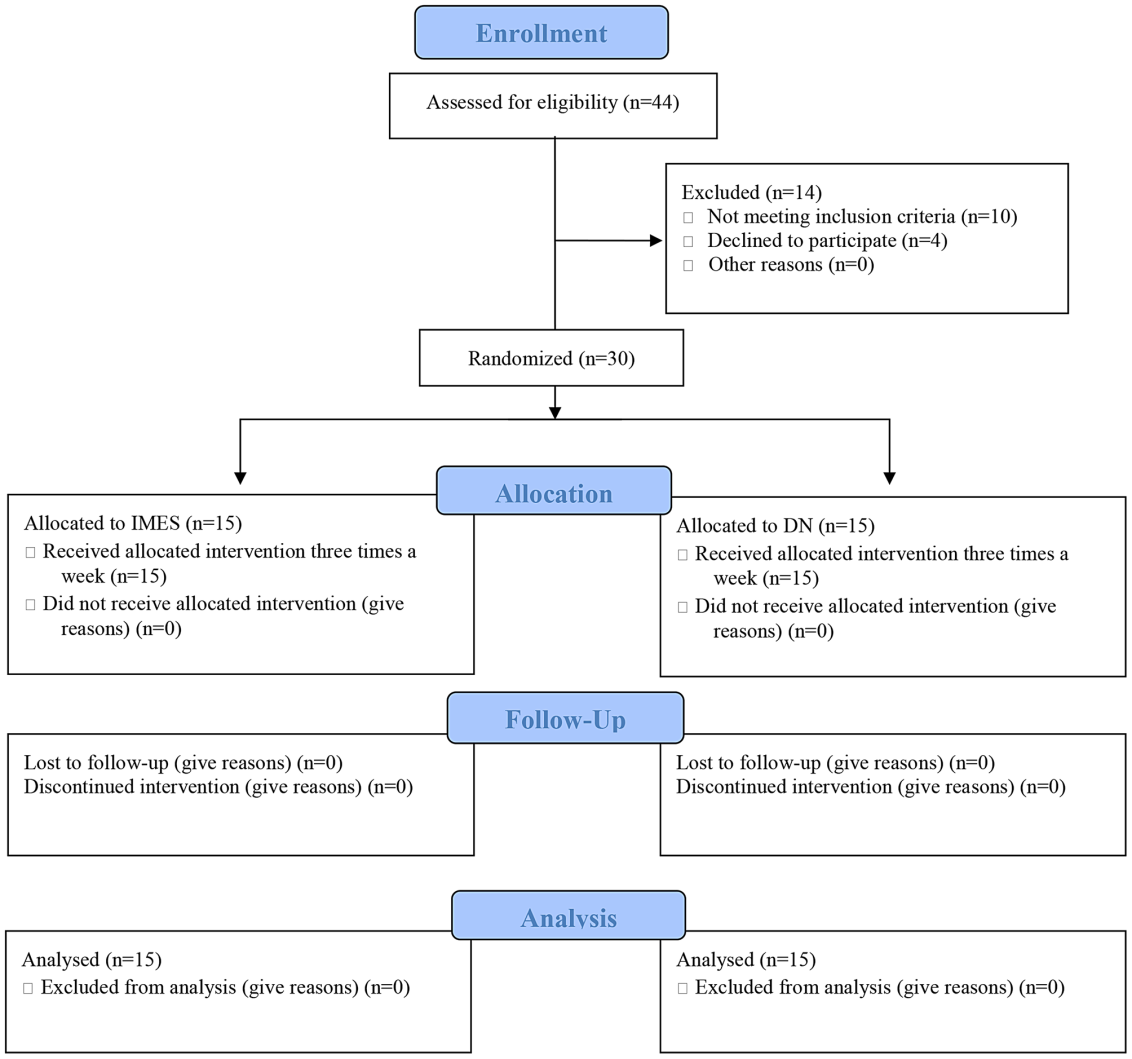
CONSORT flow diagram

44 participants were recruited. Of these, 14 were excluded, including 10 because they met the exclusion criteria and 4 because of refusal to participate. Therefore, 30 patients were included; 15 were assigned to the IMES group and 15 to the DN group. No participants dropped out during the study (Fig. 6). There were no significant differences between the two groups in terms of demographic, clinical (except NDI), and sonographic characteristics at baseline (Table 1).

**Table 1:** Demographic, clinical, and sonographic characteristics of patients

	Intervention group		*P*
	IMES (*n* = 15)	DN (*n* = 15)	
**Demographic**			
Sex (F/M)	(11/4)	(13/2)	0.33
Age (yrs.)	29.33±6.08	30±7.50	0.79
Symptom duration (month)	9.13±4.71	10.80±5.59	0.38
**Clinical**			
Neck lateral flexion	29.63±2.99	31.97±4.03	0.08
Pain intensity	61.97±15.91	58.17±16.44	0.52
Pain pressure threshold	5.09±1.34	6.23±1.99	0.07
Neck disability index	21.60±9.74	30.16±9.44	0.02
**Sonographic**			
TrP circumference	19.80±2.59	18.48±1.28	0.09
TrP longitudinal diameter	8.42±1.07	7.92±0.61	0.12
TrP transverse diameter	3.63±0.44	3.37±0.33	0.07
TrP stiffness	22.60±6.54	26.85±5.61	0.07
Blood circulation (RI)	0.74±0.09	0.67±0.11	0.06

IMES: intramuscular electrical stimulation; DN: dry needling; F: female; M: male; TrP: trigger point; RI: resistance index

**Table 2:** Primary outcome measurements between group comparisons

	Intervention group		MD	P	SMD
	IMES (*n* = 15)	DN (*n* = 15)			
**Neck lateral flexion**					
After	38.44±3.48	35.37±3.71	5.02 (2.51, 7.55)	0.000	2.10
One-month	40.29±2.99	34.90±5.12	7.36 (4.84, 9.88)	0.000	3.08
**TrP circumference**					
After	15.65±1.26	17.32±1.54	-2.41 (-3.95, -0.88)	0.000	-1.65
One-month	14.59±2.46	17.65±1.44	-3.81 (-5.34, -2.28)	0.000	-2.61

IMES: intramuscular electrical stimulation; DN: dry needling; MD: mean difference (IMESmean - DNmean); SMD: standardized mean difference

### Primary outcome measures

#### Neck lateral flexion

**Fig. 7:**
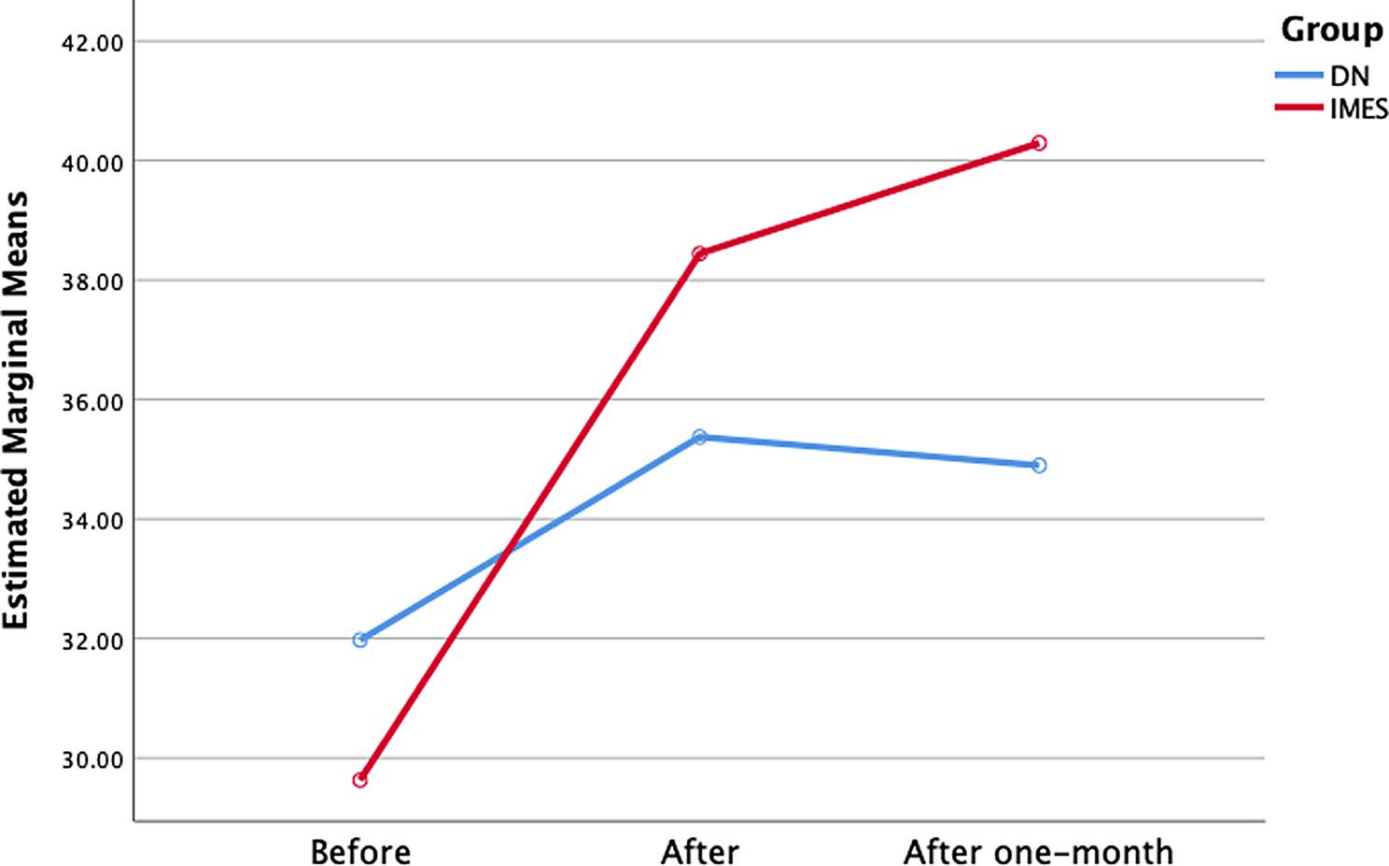
Neck lateral flexion ROM changes in both group

The ANOVA showed a significant effect on time (F = 47.13; *P* = 0.000) and interaction between group and time (F = 23.70; *P* = 0.000) also for group (F = 57.39; *P* = 0.000) for changes in neck ROM. Post hoc analysis showed that the IMES group exhibited greater ROM post-intervention than the DN group (*P* = 0.000). Furthermore, one month after intervention, the IMES group showed greater ROM than the DN group (*P* = 0.000) (Table 2; Fig. 7).

Statistically significant intra-group differences were found in both IMES and DN groups post-intervention and one month of follow-up (*P* < 0.02). Minimal clinically important difference for lateral flexion had been reported 5 degrees [62] which mean changes in IMES group were more than this measure.

#### TrP circumference

**Fig. 8:**
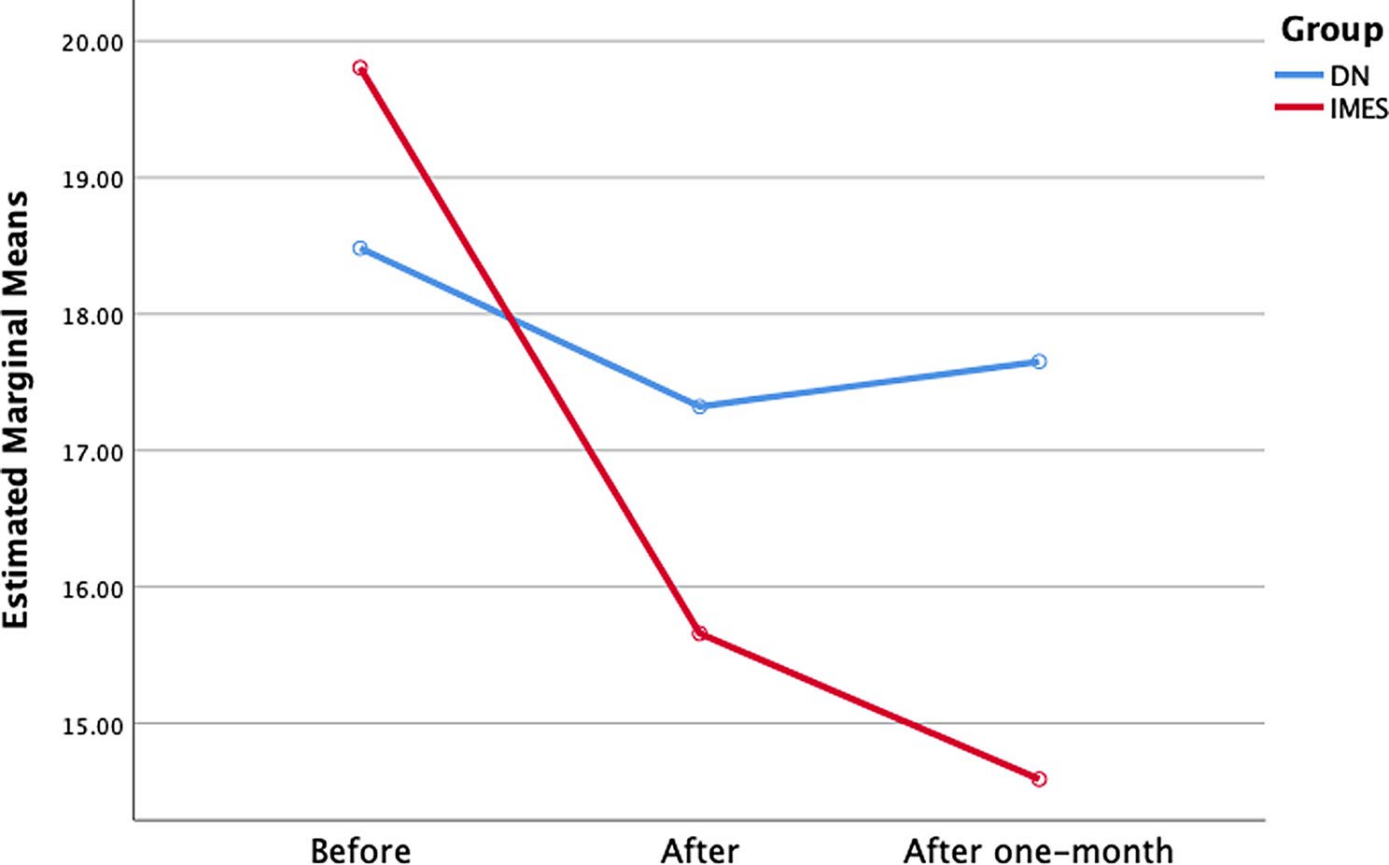
TrP CIR changes in both group

The ANOVA showed a significant effect on time (F = 24.26; *P* = 0.000) and interaction between group and time (F = 17.57; *P* = 0.000) also for group (F = 34.54; *P* = 0.000) for changes in TrP CIR. Post hoc analysis showed that the IMES group exhibited lower TrP CIR post-intervention than the DN group (*P* = 0.000). Furthermore, one month after intervention, the IMES group showed a lower TrP CIR than the DN group (*P* = 0.000) (Table 2; Fig. 8).

Statistically significant intra-group differences were found in the IMES group post-intervention and one-month follow-up (*P* = 0.000) but not in the DN group.

### Secondary outcome measures

#### Pain intensity

The Kruskal-Wallis showed no significant difference between groups in pain reduction, although the IMES group exhibited lower pain post-intervention than the DN group. Furthermore, one month after intervention, the IMES group showed lower pain than the DN group (Table 3).

**Table 3:** Secondary outcome measurements between group comparison

	Intervention group		MD	*P*	SMD
	IMES (*n* = 15)	DN (*n* = 15)			
**Pain intensity**					
After	29.77±18.99	33.37±19.57	-	0.57	-
One-month	20.21±20.62	33.93±24.06	-	0.10	-
**Pain pressure threshold**					
After	6.78±1.71	6.65±3.52	-	0.44	-
One-month	7.72±2.26	7.02±4.18	-	0.22	-
**Neck disability index**					
After	9.87±6.07	19.02±9.10	-	-	-
One-month	11.27±10.73	17.06±10.99	-	-	-
**TrP longitudinal diameter**					
After	6.56±0.55	7.41±0.70	-1.13 (-1.83, -0.44)	0.000	-1.72
One-month	6.01±1.12	7.48±0.70	-1.76 (-2.46, -1.06)	0.000	-2.67
**TrP transverse diameter**					
After	3.04±0.37	3.16±0.39	-0.30 (-0.63, 0.04)	0.107	-
One-month	2.97±0.53	3.35±0.33	-0.56 (-0.89, -0.22)	0.000	-1.76
**TrP stiffness**					
After	24.69±7.03	23.98±4.42	-	0.95	-
One-month	23.18±6.55	19.28±10.77	-	0.14	-
**Blood circulation (RI)**					
After	0.57±0.11	0.69±0.08	-0.17 (-0.24, -0.10)	0.000	-2.60
One-month	0.59±0.10	0.64±0.08	-0.10 (-0.17, -0.03)	0.002	-1.47

IMES: intramuscular electrical stimulation; DN: dry needling; MD: mean difference (IMESmean - DNmean); SMD: standardized mean difference; RI: resistance index

Statistically significant intra-group differences were found in IMES and DN groups post-intervention and one month of follow-up (*P* = 0.000). Minimal clinically important difference for pain measurement by VAS had been reported 14 mm [63], which mean changes in both groups were more than this measure.

#### Pain pressure threshold

The Kruskal-Wallis showed no significant difference between groups in PPT change, although the IMES group exhibited a greater threshold post-intervention than the DN group. Furthermore, one month after intervention, the IMES group showed a greater threshold than the DN group (Table 3).

Statistically significant intra-group differences were found in IMES groups at post-intervention and one month of follow-up (*P* = 0.001) but not in the DN group. Minimal clinically important difference for PPT in upper trapezius muscle had been reported 0.62 kg/cm2 [64] which mean changes in IMES group were more than this measure.

#### Neck disability index

The ANOVA showed no significant effect on time, interaction between group and time (F = 0.33; *P* = 0.569) and for group (F = 4.22; *P* = 0.053) for changes in NDI (Table 3).

#### TrP longitudinal diameter

The ANOVA showed a significant effect on time (F = 25.84; *P* = 0.000), interaction between group and time (F = 17.90; *P* = 0.000), also for group (F = 38.03; *P* = 0.000) for changes in TrP longitudinal diameter. Post hoc analysis showed that the IMES group exhibited lower TrP longitudinal diameter post-intervention than the DN group (*P* = 0.000). Furthermore, one month after intervention, the IMES group showed a lower TrP longitudinal diameter than the DN group (*P* = 0.000) (Table 3).

Statistically significant intra-group differences were found in the IMES group post-intervention and one-month follow-up (*P* = 0.000) but not in the DN group.

#### TrP transverse diameter

The ANOVA showed a significant effect on time (F = 11.22; *P* = 0.002), interaction between group and time (F = 8.13; *P* = 0.008), also for group (F = 12.81; *P* = 0.000) for changes in TrP transverse diameter. Post hoc analysis showed that the IMES group exhibited lower TrP transverse diameter one month after intervention than the DN group (*P* = 0.000) (Table 3).

Statistically significant intra-group differences were found in the IMES group post-intervention and one-month follow-up (*P* = 0.000) but not in the DN group.

#### TrP stiffness

The Kruskal-Wallis showed no significant difference between groups in stiffness change post-intervention and one month after intervention (Table 3).

Statistically significant intra-group differences were not found in groups at post-intervention and one month of follow-up.

#### Blood circulation

The ANOVA showed a significant effect on time (F = 12.55; *P* = 0.001) and interaction between group and time (F = 17.04; *P* = 0.000), also for group (F = 31.52; *P* = 0.000) for changes in blood circulation. Post hoc analysis showed that the IMES group exhibited higher blood circulation (lower RI) post-intervention than the DN group (*P* = 0.000). Furthermore, one month after intervention, the IMES group showed higher blood circulation (lower RI) than the DN group (*P* = 0.002) (Table 3).

Statistically significant intra-group differences were found in the IMES group post-intervention and one-month follow-up (*P* = 0.000) but not in the DN group.

## Discussion

To the best of our knowledge, this study is the first one to compare the effectiveness of IMES and DN interventions on patients with active TrP in the UT muscle using clinical and sonographic parameters. The present study showed that both interventions resulted in an equal reduction of pain intensity and PPT. The IMES intervention increased ROM significantly at both assessment times more than DN group. Similarly, the IMES group showed significantly more improvement in TrP size parameter and blood circulation than DN group.

There is a common consensus that overuse or sustained muscle contractions may lead to the formation of TrPs [65]. The integrated TrP hypothesis postulated an excessive non-quantal release of acetylcholine (ACh) in the area of TrPs [66], which was confirmed in a rodent study [67]. Excessive ACh, combined with increased levels of calcitonin gene-related peptide (CGRP) and a lowered pH, lead to localized muscle contractures, which in turn, cause ischemia and hypoxia. Hypoxia triggers the release of CGRP, substance P, and other chemicals and will lower the pH, resulting in a positive feedback cycle. The effective treatment of TrPs must focus on interrupting this vicious cycle [68].

Recently it has reported that needle insertion into the TrP in rat can decrease the end plate noise (EPN), which is correspond to Ach concentration decrement [69]. The sensitizing substances are removed from the area by increased blood flow in the TrP zone. This event reduces the stimulation of the nociceptors. It reduces symptoms such as pain and tenderness [4]. As mentioned earlier, in present study the blood circulation in the IMES group has increased significantly more than the DN group. This could be the reason why IMES is more effective than DN in some parameters. In addition, a possible mechanism in reducing TrP symptoms by IMES is that peripheral opioid receptors also appear to play a role in the analgesia produced by low-frequency TENS [70]. The frequency used in this study was 2 Hz, possibly another reason for pain relief in the IMES group.

After TrP DN, endplate noise in the TrP area decreased, which may indicate a decrease in the ACh concentration, a decrease in the actin and myosin overlap, and an increase of sarcomere length, which, in turn, may increase muscle flexibility. It is a plausible contributing mechanism of increasing ROM [58, 59]. Moreover, it seems that the noise reduction is related to the presence of LTRs [71]. Some studies have shown that LTR during DN has increased the effectiveness of DN [20, 22]. IMES intervention can induce repeated local contraction, so this can be another reason for the superiority of IMES intervention for some variable changes [70].

Eliciting muscle twitches can compress glycosaminoglycan molecules and release sensitizing substances trapped by this molecule from the TrP area into the vascular system [70], which may be more prominent with IMES and thus be more effective in disrupting the vicious cycle of TrPs. On the other hand, electric currents can cause sudden depolarization and discharge of ACh in the area. In this condition, all available synaptic vesicles are released, the terminal nerve is empty for a while, and the amount of ACh decreases [70, 72]. This event can also lead to the breaking up of the vicious cycle, which can affect the TrP permanency.

Electrical currents that lead to muscle contractions may reduce static blood flow and hypoxia [73–76]. Increased blood flow, so oxygenating can decrease the actin-myosin overlap in TrP site and also the chemical concentration. Similarly, it could be another possible IMES mechanism to disrupt TrP positive cycle and decrease triggering nociceptors.

Four studies have examined the IMES effects on TrPs [25–28]. In a single group study, Lee and colleagues assessed the influence of electrical current through a needle placed in UT muscle and levator scapulae TrPs. They reported some promising effects in symptom improvement. They did not perform an objective examination and the study did not include a control group, but their research is consistent with our findings [25]. In another study, the effects of this intervention on pain in the thoracic region were investigated and showed beneficial effects on pain relief [26]. In 2015, Sumen and colleagues investigated the IMES effects compared with low-level laser therapy on UT TrPs and they confirmed the efficacy of IMES along with exercise, which is also consistent with the present study [27]. In another study, researchers examined the IMES efficacy compared to a placebo group and showed improvements in pain and ROM [28].

Up to now, only two previous studies have compared IMES and DN and concluded that the two interventions have similar effects on pain, ROM, and disability, which is not in line with the present study in terms of ROM. The methodological differences between these studies and the present study are the frequency and the intensity reaching the sensory threshold, which may be the reason for the difference in the results. In the present study, the intensity of the current led to contraction during IMES. This applied contraction can increase the effectiveness of this intervention compared to DN to improve symptoms like ROM. In present study, in IMES group, blood circulation has increased more than DN group. This can lead to decrement in sensitizing substances in Trp area and oxygenating which can decrease the actin-myosin overlap in TrP site. This phenomenon can be the reason for more increment in ROM in present study in compare to former studies. In addition, some studies have assessed the efficacy of DN on TrPs using ultrasound investigating size, stiffness, and blood flow [77, 78].

Previous studies examined the IMES effects on TrPs with subjective factors such as pain, ROM, and questionnaires. Most of these studies are in line with the present study and reported promising effects for IMES on the clinical symptoms in individuals with TrP. None of the previous studies studied the effects of IMES objectively. In the present study, in addition to clinical parameters, sonographic parameters such as TrP size, stiffness, and blood circulation were investigated in comparison with DN. The results show that IMES is more effective compared with DN for objective parameters such as TrP size and blood circulation and clinical parameters such as ROM.

Studies have shown that ultrasound is a reliable instrument for investigating the characteristics of TrPs, such as size, stiffness, and blood flow, and may help identify TrP [47, 50, 79]. To the best of our knowledge, the present study is the first to investigate IMES effects in comparing DN on TrPs using ultrasound and clinical parameters.

## Limitations

There were several recognized limitations to this study. The study did not attempt to control the patient lifestyle before and after the initial evaluation and interventions. Furthermore, we evaluated cervical lateral flexion ROM and TrPs circumference as primary outcome measurements. Although the assessment ways of these evaluations were selected based on previous studies reported reliability of them, we had no validation process in this study to evaluate the reliability of the primary outcome measurement assessments. These limitations need to be considered in future studies. Also, it seems necessary to examine central changes such as brain changes in future studies.

## Conclusion

The results of this study show that both IMES and DN treatments can have positive and promising effects for improving UT TrP. Intramuscular electrical stimulation is more effective for some clinical symptoms and ultrasound parameters compared to DN with more permanent effects. To examine the effects of these interventions in more detail and to compare them with other common treatments and to examine the effects of this intervention on people’s lifestyles and performance, more studies with larger sample sizes are necessary.

## Electronic supplementary material

Below is the link to the electronic supplementary material.

Supplementary Material 1

## Data Availability

All data generated or analyzed during this study are included in this published article. More information is available from the corresponding author on reasonable request.

## Declarations

### Suppliers

Digital algometer. (FG-8020 -Taiwan).

Dry needle (Dong Bang 30*50).

Electrical stimulation (ES-160. by ITO (Japan)).

Goniometer

Sonography device. (Aixplorer Supersonic Imagine, France, 2017).

## Abbreviations

MPS: Myofascial pain syndrome
TrP: Trigger points
UT: Upper trapezius
DN: Dry needling
APTA: American Physical Therapy Association
LTR: Local twitch response
IMES: Intramuscular electrical stimulation
ROM: Range of motion
ANOVA: Analysis of variance
CONSORT: Consolidated Standards of Reporting Trials
VAS: Visual Analogue Scale
CIR: Circumference
PPT: Pain pressure threshold
NDI: Neck disability index
ROI: Region of interest
PSV: Peak systolic velocity
EDV: End diastolic velocity
RI: Resistance index
CI: Confidence interval
SMD: Standardized mean difference
Ach: Acetylcholine
CGRP: Calcitonin gene-related peptide
EPN: End plate noise
