# Supplementary material for: A comparative study of sonographic and clinical parameters in patient with upper trapezius muscle trigger point following dry needling and intramuscular electrical stimulation: a randomized control trial
Source: Chiropr Man Therap. 2025 Apr 14;33:14. doi: 10.1186/s12998-024-00567-8 (PMC11998240; doi:10.1186/s12998-024-00567-8)

**Declarations**

- **Ethics approval and consent to participate**

The study design was approved by the Shahid Beheshti University of Medical science Ethics Committee (IR.SBMU.RETECH.REC.1399.480, dated: August 9, 2020).

- **Consent for publication**

Subjects signed a written informed consent for publication and use their blind picture.

- **
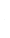
Availability of data and materials**

All data generated or analyzed during this study are included in this published article. More information is available from the corresponding author on reasonable request.

- **Competing interests**

The authors declare that they have no competing interests.

- **Funding**

This research did not receive any specific grant from funding agencies in the public, commercial, or not-for-profit sectors.

- **Authors’ contributions**
- Monavar Hadizadeh: conceptualization; data curation and extraction; investigation; methodology; project administration; resources; writing original draft; writing review and editing final draft.
-
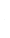
Abbas Rahimi: conceptualization; methodology; supervision; writing review and approve final draft.
- Meysam Velayati: conceptualization; data curation and extraction; methodology; writing review and approve final draft.
- Mohammad Javaherian: formal analysis; methodology; writing review and approve final draft.
- Farokh Naderi: conceptualization; methodology; supervision; writing review and approve final draft.
- Abbasali Keshtkar: formal analysis; methodology; writing review and approve final draft.
- Jan Dommerholt: conceptualization; methodology; supervision; writing review and approve final draft.
- **Acknowledgements**

The present article is part of a PhD thesis and was partially supported by the school of rehabilitation, Shahid Beheshti University of Medical Sciences.


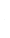


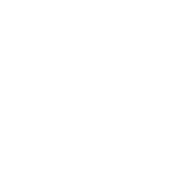

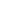

Supplement: Supplementary file 1 — Supplementary Material 1 [file 12998_2024_567_MOESM1_ESM.docx]
